# Applying Different Machine Learning Techniques for Prediction of COVID-19 Severity

**DOI:** 10.1109/ACCESS.2021.3116067

**Published:** 2021-09-28

**Authors:** Safynaz Abdel-Fattah Sayed, Abeer Mohamed Elkorany, Sabah Sayed Mohammad

**Affiliations:** Department of Computer ScienceFaculty of Computers and InformationLuxor University Luxor 85951 Egypt; Faculty of Computers and Artificial IntelligenceCairo University 63526 Cairo 12613 Egypt

**Keywords:** Chest X-rays, COVID-19, deep learning, handcrafted techniques, machine learning, mortality prediction, severity prediction

## Abstract

Due to the increase in the number of patients who died as a result of the SARS-CoV-2 virus around the world, researchers are working tirelessly to find technological solutions to help doctors in their daily work. Fast and accurate Artificial Intelligence (AI) techniques are needed to assist doctors in their decisions to predict the severity and mortality risk of a patient. Early prediction of patient severity would help in saving hospital resources and decrease the continual death of patients by providing early medication actions. Currently, X-ray images are used as early symptoms in detecting COVID-19 patients. Therefore, in this research, a prediction model has been built to predict different levels of severity risks for the COVID-19 patient based on X-ray images by applying machine learning techniques. To build the proposed model, CheXNet deep pre-trained model and hybrid handcrafted techniques were applied to extract features, two different methods: Principal Component Analysis (PCA) and Recursive Feature Elimination (RFE) were integrated to select the most important features, and then, six machine learning techniques were applied. For handcrafted features, the experiments proved that merging the features that have been selected by PCA and RFE together (PCA + RFE) achieved the best results with all classifiers compared with using all features or using the features selected by PCA or RFE individually. The XGBoost classifier achieved the best performance with the merged (PCA + RFE) features, where it accomplished 97% accuracy, 98% precision, 95% recall, 96% f1-score and 100% roc-auc. Also, SVM carried out the same results with some minor differences, but overall it was a good performance where it accomplished 97% accuracy, 96% precision, 95% recall, 95% f1-score and 99% roc-auc. On the other hand, for pre-trained CheXNet features, Extra Tree and SVM classifiers with RFE achieved 99.6% for all measures.

## I. Introduction

Predicting the severity risk of any disease at an early stage is a crucial task and has many effects, like reducing the mortality rate, consuming hospital resources, and supporting doctors in their decision making.

In the current critical period, during the spread of coronavirus around the world and the increasing number of patients and deaths, the number of COVID-19 patients reached nearly 230 million while the number of deaths was 4.7 million around the world till now during writing this research, according to statistics from Johns Hopkins University [1]. The United States is the head of the countries, followed by Brazil, India, France, Russia, Italy, and many other countries. The reasons behind this growth in numbers are the high prevalence of COVID-19, late diagnosis, and lack of resources in many hospitals to absorb this pandemic. Therefore, predicting the severity risk of COVID-19 patients is a critical task and has many positive outcomes, such as providing the required health care for each patient according to his severity, good consumption of hospital resources that give the highest priority to the high-risk patient, and assisting doctors in making their decisions that will lead to improvement in the patient’s treatment.

Three main resources that could be used to detect COVID-19: X-ray images, computed tomography (CT) and reverse transcription-polymerase chain reaction (RT-PCR). The best type is RT-PCR, but it is very expensive, not available in all hospitals and takes a lot of time to get the results. Therefore, many doctors depend on chest radiological imaging such as X-rays and CT for early diagnosis and treatment of this disease [2]. CT is a very sensitive tool, but its results can be observed after a long time according to the onset of symptoms, where normal CT takes from zero to two days to see its findings [3], so CT is difficult to use in monitoring patients periodically. Chest-X ray (CXR) radiography is less sensitive than CT and RT-PCR, but it is one of the most commonly used and accessible methods for rapid examination of lung conditions. X-ray findings are observed in a short time and it is not an expensive technique, so it can be used periodically to monitor the patient’s status.

Feature extraction is a big challenge, especially when the dataset size is small. The features of an image are quantitative data values or pixel intensities that hold meaningful information about pixels of an image in terms of local and/or global variations. It is the process of locating a feature vector representation of input images, and effectively isolating the most critical variables relevant to the aim of the intended application. Currently, two main approaches are used for feature extraction in X-ray images, namely non-handcrafted (deep learning) and handcrafted feature extraction.

The deep learning technique extracts local features from images as much as possible, but it is more suitable for large datasets. Most of the published research that has relied on chest X-ray images in its work so far focuses either on the diagnosis the disease itself or the distinction between COVID-19 and other types of pneumonia, as in [4]–[9]. These studies depended on the Convolutional Neural Network (CNN) techniques and different pre-trained models like ResNet, DenseNet, CheXNet, Xception, VGG, and others. In [10], the authors used X-ray images to detect specific severity scores of COVID-19 as a regression problem with a pre-trained deep learning model called DenseNet where X-ray images were scored retrospectively by experts in terms of the extent of lung involvement, which is called geographic extent score (range 0–8), as well as the degree of opacity, which is named lung opacity score (range 0–6), and mean absolute error (MAE) was calculated to evaluate the model. Unfortunately, sometimes deep learning models may suffer from over-fitting problems [11], cause high bias because they extract unknown and abstract features [12], and need high-dimensional datasets to obtain higher performance. To overcome such problems, some researchers used pre-trained transfer learning models to take advantage of the potential of deep learning techniques.

On the other hand, the handcrafted techniques manually extract more known or meaningful features designed for a specific problem. The important advantages of this approach are that it does not require a large dataset as well as the extraction of more related features. Examples of these papers are [13]–[16], in which the authors built a detection model to identify COVID-19 disease from other types of pneumonia based on X-ray images and used a combination of handcrafted methods to extract the features, then applied a machine learning algorithm for the detection task, where the results are promising.

Selecting the most effective image features is an important step that has a significant impact on the quality and performance of the prediction model. It aims to remove all noise, redundant, and interrelated features. Finally, only the most informative features will be retained. The outcome of this step will come back to the high performance of classifiers because the number of features has become smaller and more informative. There are a lot of methods for feature selection, such as Filter-based [17], Forward Selection, Backward Elimination, Recursive Feature Elimination (RFE), Principal Component Analysis (PCA), Linear Discriminant Analysis (LDA) [18], [19], and optimization techniques [20]–[22]. Some of these techniques were utilized for selecting features from X-ray images [23], [24] for Covid-19 detection.

X-ray images have important characteristics where they are used as the initial step for disease detection and to monitor the patient’s condition in both the hospital and ICU. For this reason, they are used in most of the current research work to detect and diagnose diseases, but, to our knowledge, not used in any research work for severity prediction. Therefore, in this study, a prediction model has been built to predict different types of severity risks of a patient based on a public dataset of X-ray images [25]. The proposed model can predict early the dangers of death and severity risks of the patient to determine the resources required to deal with the patient’s condition. It may predict whether the patient will need to enter the Intensive Care Unit (ICU) or not, as well as report his death. The model is designed to predict different levels of patient severity using handcrafted and pre-trained CheXNet techniques to extract the features of images. Then, merge the features produced by Principal Component Analysis (PCA) and Recursive Feature Elimination (RFE). Finally, classical and ensemble machine learning algorithms are applied.

The paper is organized as follows, Section II presents the material and methods of the proposed framework. Then, Section III demonstrates the experimental setup details and Section IV shows the results and discussion. After that, Section V reveals the limitations of the proposed study. Finally, Section VI concludes the study.

## II. Materials and Methods

The details of the proposed framework for COVID-19 severity level prediction are presented in this section. First, the overall architecture of the framework is described, then the applied methodology for predicting the severity level is discussed.

### A. The Proposed Architecture

**FIGURE 1.**
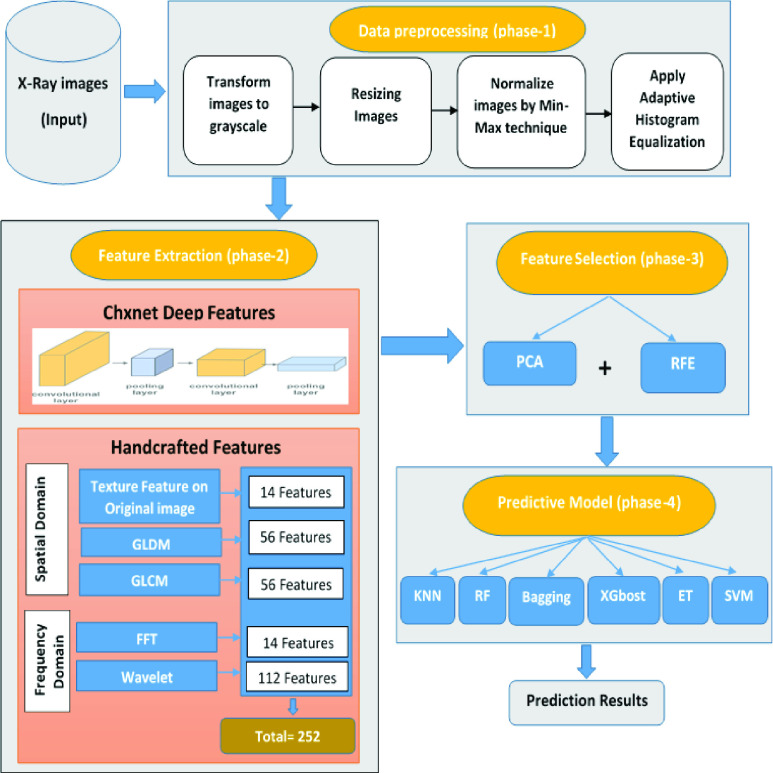
The proposed architecture.

The proposed framework consists of four main phases, as shown in Fig. 1. Firstly, the input X-ray dataset is passed to data pre-processing to resize and normalize the images. Then, different feature extraction methods are applied to extract the features. After that, feature selection techniques are executed to select the most important features in the images, and finally, different machine learning classifiers are applied to build the models.

### B. Proposed Methodology

The main phases of the proposed framework are briefly described in this section.

#### 1) Phase-1 (Data Preprocessing)

**FIGURE 2.**
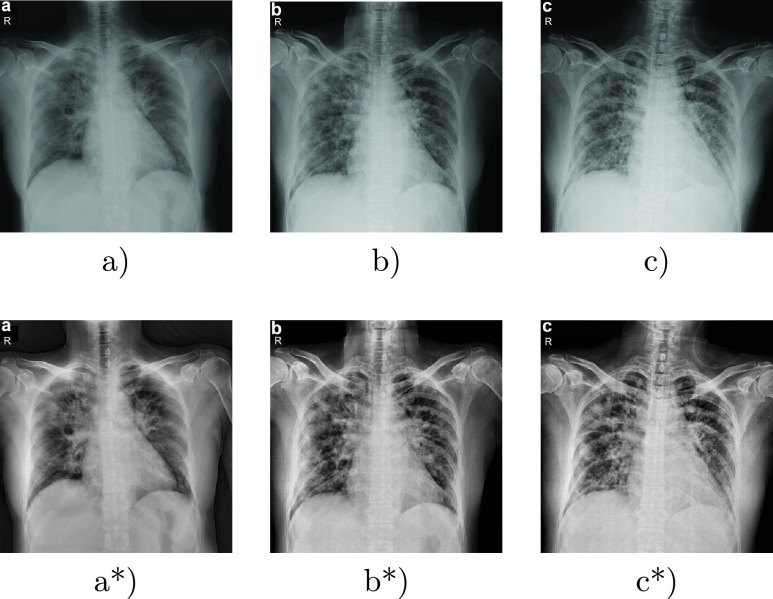
(a), (b), and (c) are examples of images before applying the preprocessing methods, while (a*), (b*), and (c*) are examples of images after applying the preprocessing methods.

The data pre-processing phase aims at preparing the data to be used in the prediction model. Usually, data are messy and come from different sources with different sizes and resolutions. So, this phase is crucial for cleaning up and normalizing the data to reduce the complexity and increase the accuracy of the prediction model. Different types of transformations could be executed according to the dataset like, re-sizing, rotating, shifting, normalizing, and so on. The four steps of preprocessing are sequentially executed on the dataset as described in Fig. 1. Firstly, transform the image to grayscale to better display diagnostically important information and optimize the radiographic information content [26]. Secondly, for handcrafted features, resize the image to $512\times 512$ dimension as recommended by [16], [27], [28] to guarantee that the image retains the most informative information about the patient’s severity level. Otherwise, being smaller than $512\times 512$ may lead to a loss in information related to the same assigned class of the whole image. For deep features, resize images to $224\times 224$ to be suitable for the required input for the CheXNet pre-trained model. Third, normalizing the image by min-max technique to rescale the image pixels in the range of 0–1, then applying Adaptive Histogram Equalization (AHE) to enhance the image contrast and improve the medical image. Fig. 2 shows examples of X-ray images before and after the data preprocessing phase.

#### 2) Phase-2 (Feature Extraction)

In this study, due to the small size of the used dataset and the motivating results of using the handcrafted techniques and pre-trained models for extracting features from medical images in other published papers [8], [9], [13], [14], [29]–[31], so the features would be extracted using two different techniques: the pre-trained CheXNet deep model and a set of handcrafted descriptors.

**FIGURE 3.**
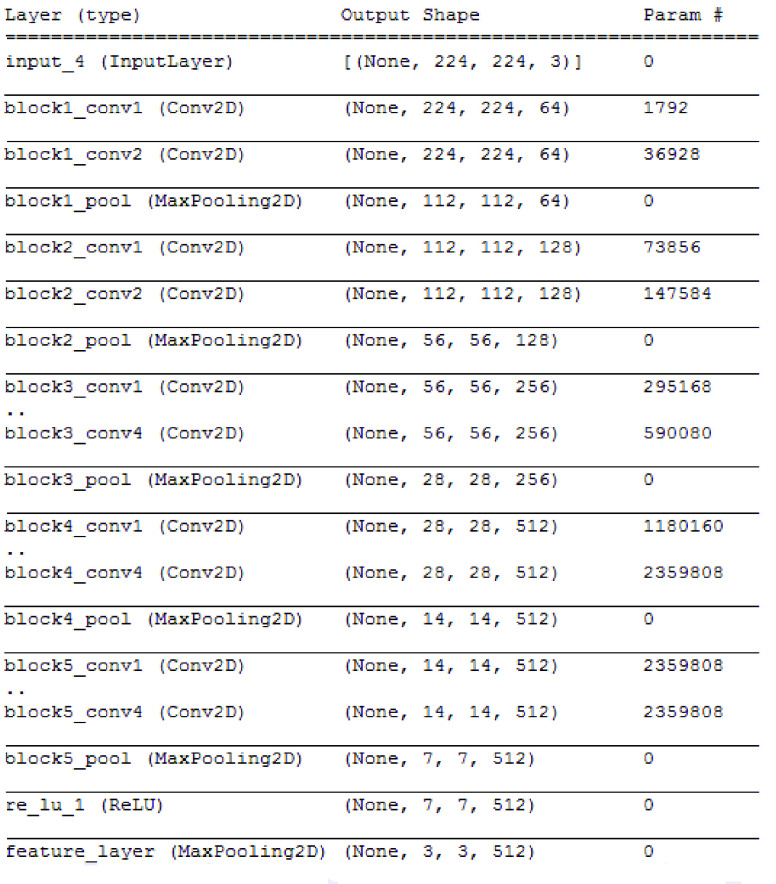
The summary design of CheXNet model.

CheXNet Deep Features:

CheXNet [32] is a 121-layer convolutional neural network based on the DenseNet architecture [33]. It trained over 100 000 frontal view chest X-ray images for 14 pneumonia diseases. The performance of CheXNet was compared with four academic radiologists who annotated a test set and is said to exceed average radiologist performance in detection. The summary design of the used pre-trained CheXNet model is outlined in Fig. 3. It consists of five convolution blocks and a max-pooling layer to extract features from X-ray images. The model generates 9216 features used by the proposed prediction model.

Handcrafted Features:

The features are extracted in the spatial and frequency domains as described in Fig. 1. The spatial domain deals with the original matrix of the image. Three methods were executed to extract features in this domain: texture features of the original image, Gray-Level Co-Occurrence Matrix (GLCM), and Gray Level Difference Matrix (GLDM).

1) Texture features of the original image: 14 features are computed (Area, Mean, Std, Skewness, Kurtosis, Energy, Entropy, Max, Min, Mean Deviation, Median, Range, Root Mean Square (RMS), and Uniformity) from the original matrix of the image.
2) Gray-Level Co-Occurrence 2. Matrix (GLCM): according to this model, co-Occurrence matrices were built in four directions (e.g., 0°, 45°, 90° and 135°), therefore the cartesian product of the 14 texture features in four different directions is applied and yields 56 features.
3) Gray Level Difference Matrix (GLDM): the same steps of GLCM were executed, but the difference matrices were built in the four directions, and then, another 56 features were extracted.

The frequency-domain deals with the rate of pixel value change in a spatial matrix domain. Two transforms were applied for feature extraction: Fast Fourier Transform (FFT) and Wavelet transform.

1) Fast Fourier Transform (FFT) is a technique for transforming digital images into a series of sine and cosine waves with different frequencies and amplitudes. The transformed content varies according to the different frequency ranges, where the higher frequency ranges have more significant texture components. Then, the same texture features mentioned above are extracted from the transformed image.
2) Wavelet transform is a technique for transforming images into time and frequency representations. Practically, it uses low-pass and high-pass filters for the high and low scales to decompose each picture into four components: approximation (I_LL_), horizontal (I_LH_), vertical (I_HL_), and diagonal (I_HH_) coefficients. After that, the 14 texture features were extracted from the transformed coefficients.

Finally, all the texture features that were extracted by the mentioned methods are combined to form the image features.

#### 3) Phase-3 (Feature Selection)

Two different methods are used for selecting the most significant features: Principal Component Analysis (PCA) and Recursive Feature Elimination (RFE). They are fast, popular in many domains, have fewer parameters, simple implementation, and require low computations. Principal Component Analysis (PCA) is an orthogonal transformation that converts a group of possibly correlated features into a smaller number of interrelated features called principal components [34]. The objective of PCA is to reduce the dimensionality of the dataset while retaining most of the original variability in the data. It is done by projecting the original dataset into the reduced space of PCA using the eigenvectors of the correlation/covariance matrix. The resulting projected data are linear combinations of the original data describing most of the variance in the data where the first component contains the largest amount of data variance. After that, each subsequent component has the remaining data variability as possible. Algorithm 1 presents the steps of PCA for reducing dimensionality.

Algorithm 1 **PCA for Dimentiality Reduction**
Input: Image features, the number of features.
Output: The most principal components
1: Standardize data by ${Z}$ scored to transform all variables to be in the same scale;
\begin{equation*} Z=\frac {X - \mu }{\sigma }.\tag{1}\end{equation*}
2: Compute the covariance matrix ( $\boldsymbol {A}$) of the standardized data for each two variables ${X}$ and ${Y}$;
\begin{equation*} \text {cov}(X,Y)=\frac {\sum _{i=1}^{n} (X_{i}-x)(Y_{i}-y)}{n-1}.\tag{2}\end{equation*}
3: Calculate the eigenvectors ( $\bar { \boldsymbol {v}}$) and eigenvalues ( $\lambda $) of the covariance matrix and store the eigenvalues in a descending order;
\begin{equation*} \bar { \boldsymbol {v}}(\boldsymbol {A} - \lambda \boldsymbol {I})=0.\tag{3}\end{equation*}
  where $\boldsymbol {I}$ is the identity matrix.
4: Sort the eigenvectors according to their decreasing order of eigenvalues;
5: Choose $\boldsymbol {k}$ eigenvectors with the largest eigenvalues;
6: Transform the data from the original dimensions to the reduced ones ( $\boldsymbol {k}$) represented by the principal components;
7: **return** the most $\boldsymbol {k}$ principal components.

Recursive Feature Elimination (RFE) [35] is a backward wrapper feature selection algorithm that uses a machine learning classifier to select the optimal features. The classifier is used as an objective function and RFE tries to find the most effective features that increase the classification result. The process of RFE for feature selection works as presented in Algorithm 2:

Algorithm 2 **RFE for Feature Selection**
Input: Image features, the number of features $N$, supervised classifier.
Output: The best selected features.
1: **while** ( $n != N$) **do**
2: Fit the classifier with all features;
3: Rank features according to its importance for the model results;
4: Remove the weakest feature;
5: **if** the desired number of features are reached, stop.
**else,**
go to step 2;
6: **end while**
7: **return** the best selected features.

#### 4) Phase-4 (Prediction Models)

To build a robust predictive model, different machine learning classifiers were compared. Six classifiers: K Nearest Neighbors (KNN), Random Forest (RF), Extra Tree (ET), Bagging, eXtreme Gradient Boosting (XGBoost), and Support vector machine (SVM) were used.

K Nearest Neighbors (KNN) is a supervised algorithm developed by Thomas Cover [36] for classification and regression problems. It uses a feature similarity method to predict the label of a new given point, which further means that the new test point will be classified according to the majority vote of the nearest ${K}$ neighbours in the training set, where ${K}$ is the number of neighbours. It is characterized as simple, easy-to-implement, depending on only a single parameter ( ${K}$), and effective classifiers.

Bootstrap aggregating, also known as (Bagging), is one of the simplest ensemble-based techniques that was developed by Breiman [37]. It is used for variance reduction for those algorithms that have high variance, like decision tree classifiers. It is designed to make decision trees more robust and to achieve better performance. The idea behind the bagging classifier is to gather the predictions of several “weak learners” to form a “strong learner” with a more accurate output. Here, each decision tree refers to a “weak learner”, whereas a combination of these trees together is a “strong learner”. It uses the bootstrap concept to generate new training sets from the original dataset with replacements, where the generated dataset is called the Bootstrapped dataset. The size of the bootstrapped dataset is typically the same or smaller than the size of the original dataset. The steps of the classifier are as following:

1) Sample many new random training sets from the original dataset with replacements (Bootstrapped datasets);
2) Build a decision tree classifier for each created bootstrapped dataset;
3) Feed the original dataset into each of the previously built classifiers and keep track of the determined classification;
4) Use the determined classification to select the final decision by majority voting.

The Random Forest (RF) is an ensemble classifier and an improved version of the original bagging classifier. The forest consists of many decision trees and the final classification result is determined by aggregating all classification results of these composed trees and taking the majority vote of classification results from them [38]. It differs from the bagging algorithm by using a learning algorithm that selects a random subset of the features at each split in the growing phase. The reason for doing this is to make all the decision trees different because each tree uses a random different subset of data.

Extra Tree (ET) is a supervised ensemble classifier proposed by Geurts *et al.* [39]. It is also based on a set of decision trees, uses majority voting for the final decision on classification problems, and randomly selects a subset of features when choosing the partition of each node like RF. The difference between ET and any other ensemble algorithm is that it contains a bias/variance analysis where it uses the whole original samples rather than bootstrapped samples that will reduce the bias. On the other hand, the selection of cut points to split nodes in the tree is random, which will reduce the variance. The performance is similar to other ensemble classifiers, but ET can be computationally faster [40].

EXtreme Gradient Boosting (XGBoost) is an ensemble supervised classifier [40]. It is a scalable machine learning system for gradient tree boosting. XGBoost builds one tree at a time in a forward manner; each new tree is created and added to the ensemble model to correct the errors made by the previous ones sequentially until no further improvements can be made; and then, the trees added together to predict the final result. It is named gradient boosting because it uses the gradient descent algorithm to decrease the loss when adding new trees. It differs from RF, where RF builds each tree independently, while XGBoost adds each one sequentially. Also, RF combines the results of the trees at the end of the process by voting for the majority result, whereas XGBoost combines the results along the way. The most important advantage of this classifier is that it uses a more regularized model formalization to reduce over-fitting and enhance performance.

Support vector machine (SVM) is a supervised machine learning algorithm proposed by Cortes and Vapnik [41]. The objective of SVM is to find the optimal decision boundary with a maximum margin hyperplane between the different classes of samples. To achieve that, SVM needs to convert the space of the input data from a low-dimensional space into a higher-dimensional space to separate datasets into different samples with the optimal boundary. This conversion is implemented by a technique called a kernel. The kernel converts non-separable problems into separable problems by adding more dimensions to data. The commonly used kernel methods include Radial basis kernel, Polynomial kernel, and Linear kernel.

## III. Experimental Setup

In this section, all the details of the used dataset, feature selection techniques, machine learning classifiers, and evaluation metrics are described.

### A. Dataset

A public-available dataset developed by Cohen JP [25] has been used. The dataset contains data about COVID-19 patients and other pneumonia patients. It includes information about the patient like patient-id, age, X-ray image, type of disease, survival or not, and went-ICU or not. The cohort of the paper was built by first excluding all patients less than 18 years old; selecting only the confirmed COVID-19 patients that had a positive RT–PCR test: 40% female and 60% male patients; and then, using the variables that represent patient status, such as survival or not, as well as went-ICU to classify the patients. The aforementioned variables are used to label the severity of patients. Actually, two main variables are used to identify the severity level, namely survival and went-ICU variables. The classification of the severity is done according to the following rules:

• if survival is false, it is called *high severity*;
• if survival and went-ICU are true, it is termed *moderate severity*;
• if survival is true and went-ICU is false, it is named *low severity*.

These classification rules include mortality patients with a high severity class, patients who need to enter ICU as a moderate severity class, and patients with stable conditions who do not need to enter ICU as a low severity class, where the size of the cohort is 127 images. The splitting of the dataset is done by an 80/20 percentage for train/test sets and grouped by the patient-id to ensure that all X-ray images relating to each patient are distributed to only one (train/test) set.

### B. Feature Selection Methods

#### 1) Principal Component Analysis (PCA)

**FIGURE 4.**
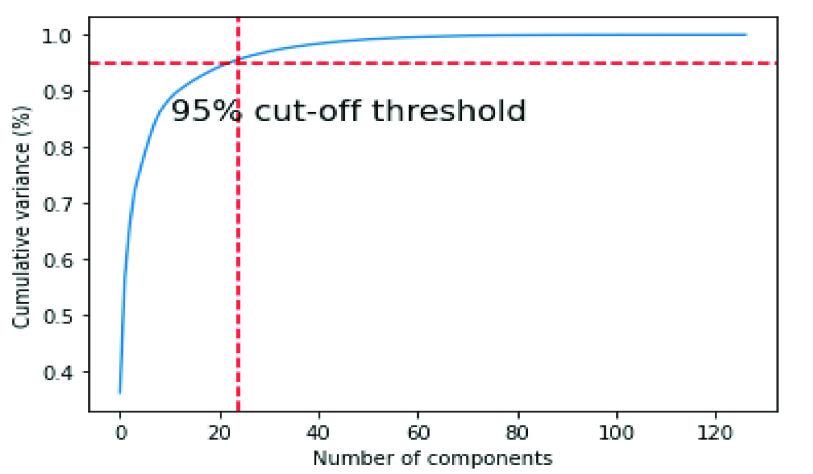
The number of PCA components for the handcrafted features.

The aforementioned PCA steps were applied to the original data. Fig. 4 shows the variance ratio of different components for handcrafted and it is noted that 24 components represent 95% of the variance of the original data, so the number of selected principal components is 24 for handcrafted features. On the other hand, the variance ratio of CheXNet components is 103 components, which represents 95% of the variance of the original features.

#### 2) Recursive Feature Elimination (RFE)

There are two types of hyperparameters required: the number of features to be selected and the algorithm to be used as an objective function in the feature selection process. Various algorithms have been tried, like gradient boosting, logistic regression, decision tree, random forest, and perceptron with different numbers of features. Every outcome of selected features by these experiments has been tested on the used machine learning classifiers in this study. Due to the large number of results, only the best hyper-parameter selection values are mentioned here, where the best estimator algorithm is perceptron and the optimal number of features is 28 for handcrafted and 100 for CheXNet deep features that performed well with the classifiers.

### C. Machine Learning Classifiers

For the hyper-parameters of the used classifiers, the grid search algorithm and a try and error approach were applied to find out the best values for them. Table 1 reports the hyper-parameter values of the used classifiers.

**TABLE 1:** Hyper-Parameters of Each Classifier

Classifier	Parameters
KNN	n_neighbors=10.
RF	n_estimators=100; max_depth=6; max_features=0.2.
XGboost	n_estimators=100; learning_rate=0.3; objective=multi:softprob; subsample=0.5.
Bagging	n_estimators=100; base_estimator=DecisionTree; max_features=0.2; max_samples=0.9.
ET	n_estimators=100; max_features=0.2; min_samples_split=15.
SVM	kernel=rbf; ${C}$=3; gamma=0.1.

### D. Evaluation Metrics

In order to evaluate the performance of the models, various scores were measured to ensure the results, like accuracy, macro-avg of sensitivity/recall, precision, f1 scores, and AUROC. The variable ${C}$ refers to the number of classes and $i \in C$ indicates to a specific class. TP, TN, FP, and FN stand for True Positive, True Negative, False Positive, and False Negative, respectively.

• The precision score measures the ratio of correctly predicted positive observations to the total predicted positive observations by (4).
\begin{equation*} \text {Precision}_{i}=\frac {\text {TP}_{i}}{\text {TP}_{i} + \text {FP}_{i}}.\tag{4}\end{equation*}
• Recall/Sensitivity calculates the ratio of correctly predicted positive observations to all observations in a true class using (5).
\begin{equation*} \text {Recall}_{i}=\frac {\text {TP}_{i}}{\text {TP}_{i} + \text {TN}_{i}}.\tag{5}\end{equation*}
• F1-score is the weighted average between Precision and Recall calculations and is computed by (6). It can be documented that this score is more useful than accuracy because it takes into account false positive and false negative measurements.
\begin{equation*} \text {F1-Score}_{i}=\frac {2 (\text {Recall}_{i} \times \text {Precision}_{i}) }{\text {Recall}_{i} + \text {Precision}_{i}}.\tag{6}\end{equation*}
• Accuracy simply calculates the ratio of correctly predicted observations to the total observations, as in (7).
\begin{equation*} \text {Accuracy}=\frac {\text {TP} + \text {TN}}{\text {TP} + \text {TN} +\text {FP} +\text {FN}}.\tag{7}\end{equation*}
• To evaluate the general performance of different classifiers, the Macro-avg score has been chosen and computed as (8) for averaging calculation by classes.
\begin{equation*} \text {Macro-avg(measure)}=\frac {\sum _{i} \text {measure}_{i}}{C}.\tag{8}\end{equation*}
• The Area Under the Receiver Operating Characteristics (AUROC) is computed. It is an important performance measurement for classification problems. It computes the capability of the model to distinguish between the different classes, which means that the model with a higher AUC value can predict the class sample as its actual value.

It is important to mention that all these measurements were computed for all different classifiers in all performed experiments.

## IV. Experimental Results & Discussion

It is worth mentioning that many experiments have been executed and, due to the large number of results, only the main experiments are presented here. The paper experiments were implemented in the Python programming language using the sklearn package with its libraries for reporting the results like classification report, confusion matrix, roc curve, and AUC. All experiments were performed on the dataset mentioned in section III-A. Firstly, the preprocessing steps and the feature extraction techniques mentioned in II-B1 and II-B2 were applied. Then, the feature selection step is performed in different ways, as follows:

1) All extracted features were used (No feature selection methods were used).
2) PCA is applied to the extracted features.
3) RFE is applied to the extracted features.
4) The features that were selected by PCA and RFE are combined together (PCA + RFE).

Finally, the machine learning classifiers mentioned in part III-C were executed. The experimental results are presented in the following tables have meaningful column names: the “All” column means using all extracted features; the “PCA” and “RFE” columns denote the results of the selected features by PCA and RFE techniques individually; and the “(PCA + RFE)” column shows the results of the combined features of PCA and RFE techniques together. This section is divided into two subsections where the first one is related to the experiments over the handcrafted extracted features and the second one displays the results of the experiments over CheXNet extracted features by applying the used classifiers.

### A. Experiments Over Handcrafted Features

Table. 2 contains the number of the used handcrafted features in each experiment.

**TABLE 2:** The Number of the Used Handcrafted Features

Experiment name	Number of features
All	252
PCA	24
RFE	28
PCA + RFE	52

**FIGURE 5.**
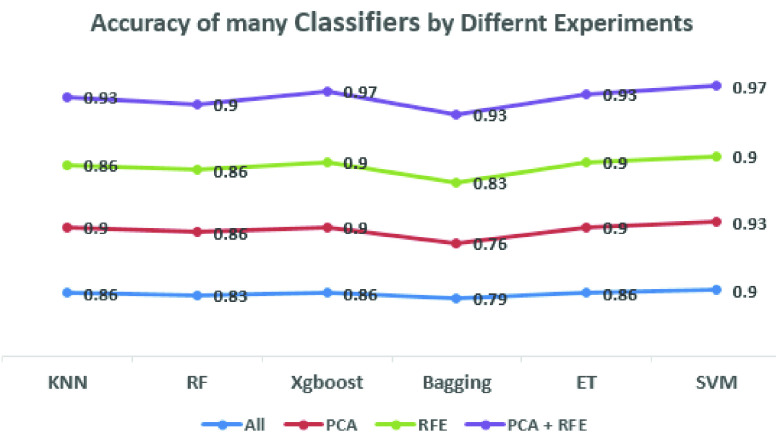
The accuracy of the used classifiers by different experiments over the handcrafted features.

The results in Tables. 3, 4, 5, 6, 7, and 8 demonstrate that using the combined features of PCA and RFE over the handcrafted extracted features achieved the best results on all scores: accuracy, precision, recall, F1-score, and Roc-AUC with all classifiers compared with using all extracted features, PCA features, or RFE features alone. Also, the findings in Tables. 3 and 8 show that using the selected features by PCA was better than using the selected features by RFE with KNN and SVM classifiers respectively. It is appeared that RFE surpassed PCA with the Bagging classifier as described in Table 6, while in the remaining results there were no big differences between using PCA or RFE with ensemble classifiers like Random Forest, XGBoost, and Extra Tree as presented in Tables. 4, 5, and 7 respectively. The compared results in Fig. 5 show that SVM and XGBoost achieved the best accuracy (97%) by using the merged features (PCA + RFE) compared with other classifiers.

**TABLE 3:** Results of the KNN Classifier Over the Handcrafted Features

Score	All	PCA	RFE	PCA + RFE
Accuracy	0.86	0.90	0.86	0.93
Precision	0.87	0.90	0.87	0.93
Recall	0.81	0.86	0.81	0.90
F1-score	0.83	0.87	0.82	0.90

**TABLE 4:** Results of the Random Forest Classifier Over the Handcrafted Features

Score	All	PCA	RFE	PCA + RFE
Accuracy	0.83	0.86	0.86	0.90
Precision	0.81	0.88	0.88	0.90
Recall	0.79	0.81	0.81	0.86
F1-score	0.80	0.82	0.82	0.87

**TABLE 5:** Results of the XGBoost Classifier Over the Handcrafted Features

Score	No selection	PCA	RFE	PCA + RFE
Accuracy	0.86	0.90	0.90	0.97
Precision	0.85	0.88	0.91	0.98
Recall	0.86	0.91	0.86	0.95
F1-score	0.85	0.89	0.88	0.96

**TABLE 6:** Results of the Bagging Classifier Over the Handcrafted Features

Score	All	PCA	RFE	PCA + RFE
Accuracy	0.79	0.76	0.83	0.93
Precision	0.82	0.79	0.81	0.93
Recall	0.74	0.67	0.79	0.90
F1-score	0.76	0.69	0.79	0.90

**TABLE 7:** Results of the ET Classifier Over the Handcrafted Features

Score	All	PCA	RFE	PCA + RFE
Accuracy	0.86	0.90	0.90	0.93
Precision	0.88	0.90	0.91	0.93
Recall	0.83	0.86	0.86	0.90
F1-score	0.83	0.87	0.86	0.90

**TABLE 8:** Results of the SVM Classifier Over the Handcrafted Features

Score	All	PCA	RFE	PCA + RFE
Accuracy	0.90	0.93	0.90	0.97
Precision	0.90	0.93	0.90	0.96
Recall	0.88	0.90	0.86	0.95
F1-score	0.87	0.90	0.87	0.95

**FIGURE 6.**
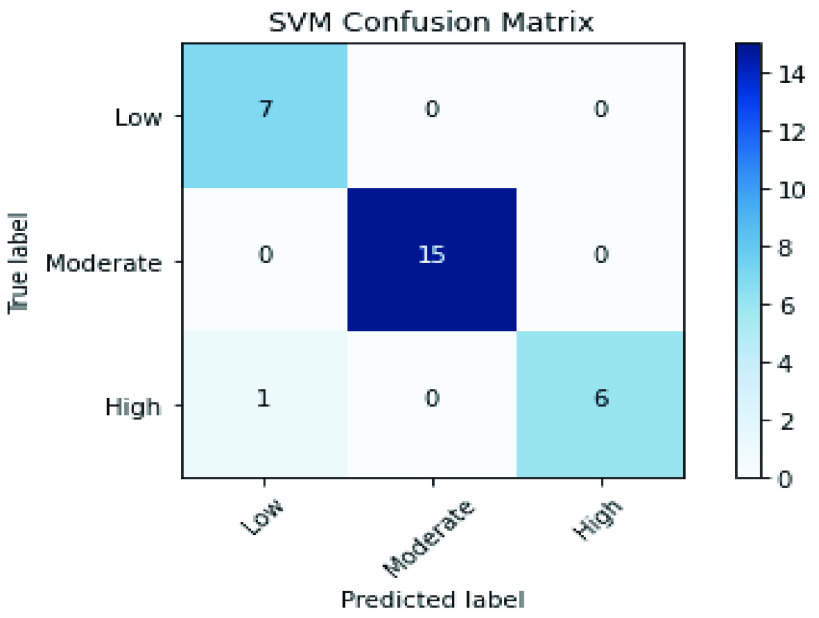
Conflusion matrix of the SVM classifier over the merged handcrafted features (PCA + RFE).

**FIGURE 7.**
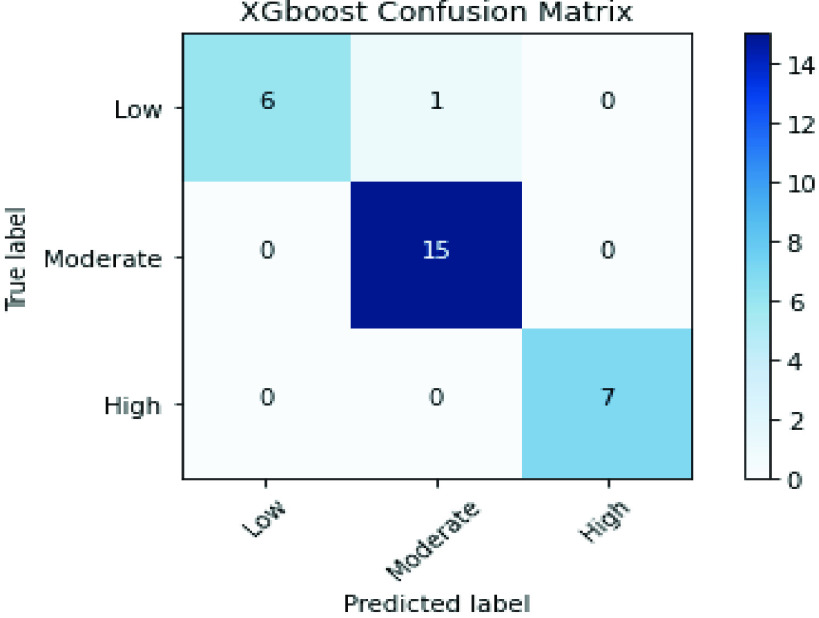
Conflusion matrix of the XGBoost classifier over the merged handcrafted features (PCA + RFE).

**FIGURE 8.**
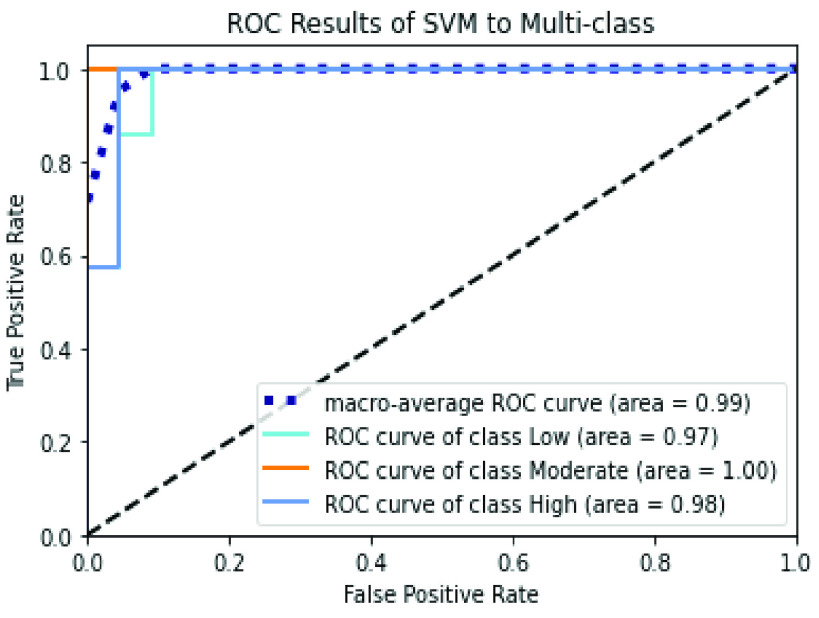
Roc curve of the SVM classifier over the merged handcrafted features (PCA + RFE).

**FIGURE 9.**
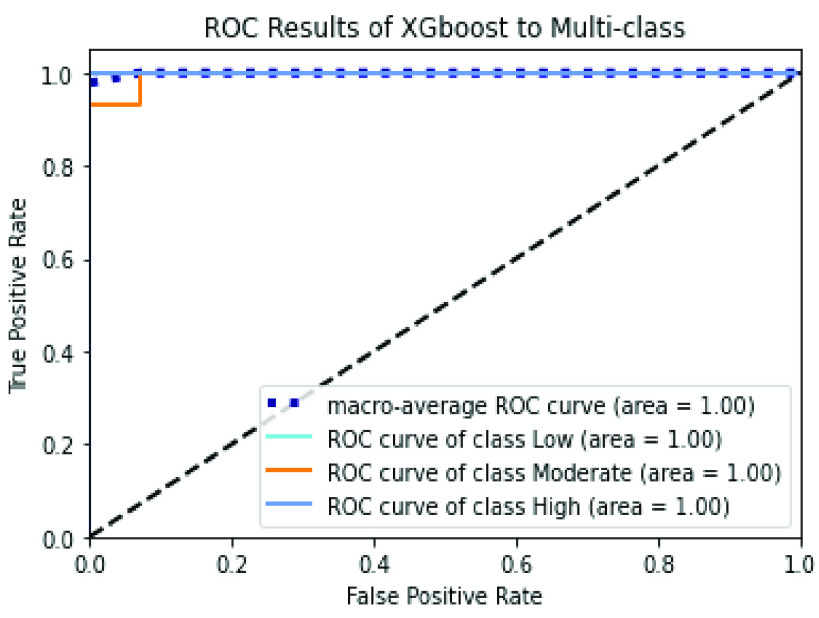
Roc curve of the XGBoost classifier over the merged handcrafted features (PCA + RFE).

The confusion matrices of SVM and XGBoost classifiers are described in Figs. 6 and 7 to show the details related to the values of True Positives (TP), True Negatives (TN), False Positives (FP), and False Negatives (FN) for each classifier. Also, The Roc-AUC results of the same classifiers are perfect for the different classes as presented in Figs. 8 and 9.

### B. Experiments Over CheXNet Deep Features

The details about the number of used CheXNet features for each experiment are described in Table 9.

**TABLE 9:** Number of the Used CheXNet Features

Experiment name	Number of features
All	9216
PCA	103
RFE	100
PCA + RFE	203

**FIGURE 10.**
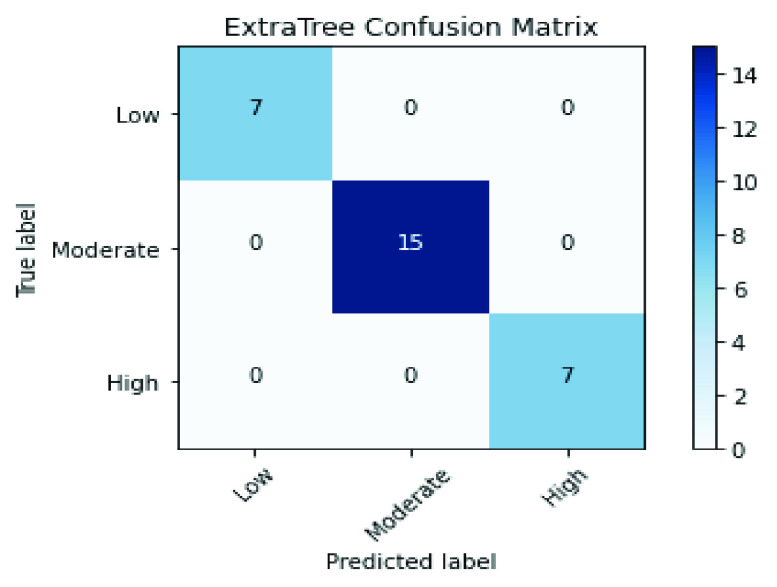
Conflusion matrix of the ET classifier by applying RFE over the CheXNet features.

**FIGURE 11.**
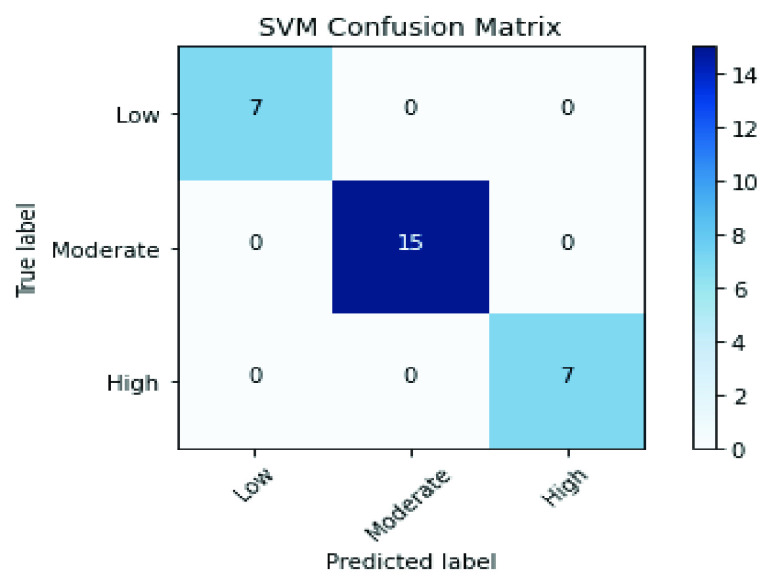
Conflusion matrix of the SVM classifier by applying RFE over the CheXNet features.

For CheXNet deep features, the results in Tables. 10, 11, 12, 13, 14, and 15 demonstrate that RFE has achieved the best results with all classifiers compared with All, PCA, or hybrid features (PCA + RFE) features. The RFE selected the optimal features from CheXNet deep features and gained nearly 99.6% for all measures with Extra Tree and SVM classifiers where their confusion matrix results, as in Figs. 10 and 11, display the perfect values of TP, TN, FP, and FN.

**TABLE 10:** Results of the KNN Classifier Over the CheXNet Features

Score	All	PCA	RFE	PCA + RFE
Accuracy	0.59	0.59	0.97	0.66
Precision	0.56	0.58	0.98	0.63
Recall	0.56	0.58	0.95	0.65
F1-score	0.56	0.58	0.96	0.64

**TABLE 11:** Results of the Random Forest Classifier Over the CheXNet Features

Score	All	PCA	RFE	PCA + RFE
Accuracy	0.86	0.69	0.93	0.90
Precision	0.90	0.80	0.96	0.94
Recall	0.81	0.62	0.90	0.86
F1-score	0.81	0.60	0.92	0.88

**TABLE 12:** Results of the XGBoost Classifier Over the CheXNet Features

Score	No selection	PCA	RFE	PCA + RFE
Accuracy	0.66	0.72	0.97	0.93
Precision	0.61	0.80	0.96	0.96
Recall	0.55	0.67	0.95	0.90
F1-score	0.54	0.69	0.95	0.92

**TABLE 13:** Results of the Bagging Classifier Over the CheXNet Features

Score	All	PCA	RFE	PCA + RFE
Accuracy	0.69	0.83	0.97	0.93
Precision	0.82	0.86	0.96	0.96
Recall	0.60	0.79	0.95	0.90
F1-score	0.59	0.80	0.95	0.92

**TABLE 14:** Results of the ET Classifier Over the CheXNet Features

Score	All	PCA	RFE	PCA + RFE
Accuracy	0.66	0.86	99.6	0.97
Precision	0.87	0.88	99.6	0.98
Recall	0.52	0.83	99.5	0.95
F1-score	0.53	0.84	99.3	0.96

**TABLE 15:** Results of the SVM Classifier Over the CheXNet Features

Score	All	PCA	RFE	PCA + RFE
Accuracy	0.66	0.69	99.6	0.69
Precision	0.73	0.77	99.7	0.77
Recall	0.57	0.62	99.5	0.62
F1-score	0.58	0.63	99.4	0.63

Finally, we can conclude that the feature extraction and selection steps are the most important steps and have the most effective effect on the results of the prediction model. Also, choosing the method of feature selection should be related to the type of the extracted features, as in the aforementioned results, where RFE has accomplished perfect results with the pre-trained CheXNet extracted features, whereas the combined techniques (RFE + PCA) have achieved promising results with the handcrafted features. Overall, these results prove that utilizing machine learning techniques in the COVID-19 pandemic would improve the healthcare system all over the world. This paper follows most of the proposed recommendations in [12], like implementation replicability, where the source code has been uploaded on GitHub at https://github.com/safynaz/Cov-Sev,. The data pre-processing methods and the techniques for every step in the prediction model have been explained in sufficient detail as described in Section II. Also, the demographics of the used dataset did not include any pediatric images, but patients less than 18 years old were excluded, where most of the used cohort started at 20 years old and were 40% female and 60% male. The paper used two different techniques for feature extraction: handcrafted and deep learning techniques, not deep learning only like in some studies, which may cause high bias and overfitting. The most interesting thing is that the results have not been compared with the RT-PCR tests as the ground truth because some papers doubt that the RT-PCR test may be negative but the patient has COVID-19 whereas the paper results are compared with the ground truth of what happened with COVID-19 patients in the three levels of severity, whether patients would enter ICU, die or not.

## V. Limitations of the Study

The target of this study is to propose a prediction model for early severity for COVID-19 patients, but the proposed models suffer from some limitations that can be solved in future work. Firstly, due to the small size of the available dataset, the proposed model used specific feature extraction techniques like deep transfer learning CheXNet and handcrafted descriptors rather than building and training deep models based on large and different types of COVID-19 severity images. Secondly, the proposed models were only validated internally and lack external validation due to data privacy and hospitals are not allowed to give any data. Third, although the prediction model based on the deep extracted features achieved perfect results with all measurements, it lacks interpretability and transparency because the extracted features are unknown and abstract over several model layers and neurons. Furthermore, most of the available images are compressed into JPEG and PNG, not the original DICOM formats, causing a loss in image quality and a lack of consistency.

## VI. Conclusion

This study proposes a new predictive framework for the severity and mortality risk of COVID-19 patients to help doctors, hospitals, and medical facilities in their decision making about which patients need to get attention first before others, and at the same time, to keep hospitals’ resources for high-risk priority patients. The proposed model is based on a public X-ray image dataset for confirmed patients with COVID-19 disease. The dataset is classified into three severity classes: high, moderate and low severity labels. The high severity class means that a patient may die, while the moderate severity class refers that a patient will need to enter the ICU, whereas the low severity indicates that a patient will not need to enter the ICU. Pre-trained deep CheXNet and hybrid handcrafted techniques were applied to extract the features from X-ray images, then two feature selection techniques were merged together: PCA and RFE, and many predictive models were built based on machine learning algorithms like KNN, Random Forest (RF), XGboosting, Bagging, Extra Tree, and SVM to compare and ensure the results. Many experiments were executed and the results revealed that, for handcrafted features, merging the selected features by PCA and RFE (PCA + RFE) achieved the best results with all classifiers where the number of used features is 52, which is nearly 25% of the original number of extracted features (252). Also, XGboost and SVM surpassed other classifiers with an accuracy of 97% and 100% roc-AUC with (PCA + RFE) selected features. On the other hand, for CheXNet deep features, RFE has achieved promising results with all measures by all classifiers and 99.6% for all measures with SVM and Extra tree classifiers where the number of selected features is 100 from originally 9216 features.
